# Agouti-related protein as the glucose signaling sensor in the central melanocortin circuits in regulating fish food intake

**DOI:** 10.3389/fendo.2022.1010472

**Published:** 2022-11-01

**Authors:** Juan Han, Xiaofang Liang, Yanzhi Guo, Xiaoliang Wu, Ziqi Li, Tiannuo Hong

**Affiliations:** ^1^ Institute of Food and Nutrition Development, Ministry of Agriculture and Rural Affairs, Beijing, China; ^2^ Feed Research Institute, Chinese Academy of Agricultural Sciences, Beijing, China; ^3^ Department of Research Management, Chinese Academy of Agricultural Sciences, Beijing, China

**Keywords:** Agouti-related protein, AgRP, glucose signaling sensor, MC3R, MC4R, POMC, food intake

## Abstract

Agouti-related protein (AgRP) is a neuropeptide synthesized by AgRP/NPY neurons and transcribed as 132 amino acids in humans and 142 amino acids (AgRP1) in Japanese seabass (*Lateolabrax maculatus*) fish. AgRP neurons are activated by hormonal signals of energy deficits and inhibited by signals of energy surpluses and have been demonstrated to have the ability to sense the dynamics of blood glucose concentrations as the “glucose sensor” in mammals. It is widely recognized that AgRP is an endogenous antagonist of the melanocortin-3 and -4 receptors (MC3R and MC4R) in the hypothalamus, exhibiting potent orexigenic activity and control of energy homeostasis. Most fish, especially carnivorous fish, cannot make efficient use of carbohydrates. When carbohydrates like corn or wheat bran are added as energy sources, they often cause feeding inhibition and metabolic diseases. When fishmeal is replaced by plant protein, this does not completely eliminate carbs, limiting the utilization of carbohydrates and plant proteins in aquaculture. Our previous study showed that AgRP, and not neuropeptide Y (NPY) is the principal protein molecule that correlates well with feeding behavior in Japanese seabass from anorexia to adaptation. The Ghrelin/Leptin-mTOR-S6K1-NPY/AgRP/POMC feed intake regulatory pathway responds to the plant-oriented protein which contains glucose. However, its regulatory function and mechanism are still not clear. This review offers an integrative overview of how glucose signals converge on a molecular level in AgRP neurons of the arcuate nucleus of the hypothalamus. This is in order to control fish food intake and energy homeostasis.

## Introduction

Agouti-related protein (AgRP) is a neuropeptide synthesized by the brain’s AgRP/NPY neurons (1). It is only synthesized in the cell body containing NPY in the ventral part of the arcuate nucleus of the hypothalamus (2, 3). Recently, a large number of studies have demonstrated that AgRP is a long-acting appetite-stimulating signaling factor that regulates appetite, food intake (4–7), energy metabolism and balance (1, 8–13), and glucose homeostasis (12, 14–16). Therefore, it is related to anorexia (17–19), obesity (2, 20–22), and different kinds of metabolic diseases (17, 23–27).

Many hormones implicated in the control of glucose homeostasis have been shown to affect either AgRP mRNA expression or AgRP neuron excitability, including insulin (28–30), leptin (14, 31), and ghrelin (17, 32). It has also been demonstrated that nutrients, such as glucose (33), amino acids (34, 35), and fatty acids (36), play an important role in the regulation of AgRP neuron activity. Blood glucose concentrations fluctuate depending on physical activity or meal intake (37). Specific neuronal populations can sense the dynamics of blood glucose concentrations in the body (15), and the mechanisms underlying this effect have been well-defined over the decades (12, 14–16, 28–30, 33).

Glucose can be actively transported across the blood–brain barrier. Depending on the brain area, differential tightness of the local blood–brain barrier can enhance or decrease the ability of neurons to sense acute changes in glycemia (15, 31). Partially due to their position close to the median of the brain, Arc neurons, including AgRP/NPY neurons and pro-opiomelanocortin (POMC) neurons, are well known to respond to changes in surrounding glucose concentrations or metabolic hormones (insulin, leptin), either as being glucose excited or inhibited (8, 38–41).

Although previous studies have shown that AgRP is a small endogenous glucose signaling molecular sensor within the hypothalamus and exhibits potent orexigenic activity and control of energy homeostasis, its regulatory function and mechanism are still unclear. In this review, we summarized the latest knowledge on the mechanisms of action and function of AgRP in regulating glucose sensing and metabolism. We also compared this with the effects on food intake in fish. We hope to provide a new strategy for solving carbohydrate energy sources’ utilization and the replacement of plant protein in fishmeal used in aquaculture cultivation. This review offers an integrative overview concerning how glucose signals converge on a molecular level in AgRP neurons of the arcuate nucleus of the hypothalamus to control fish food intake and energy homeostasis.

## AgRP neuropeptide and the brain, the hypothalamus, and the neurons

In neuroscience, a longstanding goal of the field is to understand the relationship between brain function and behavioral states such as hunger (42–44). Appetite is controlled by multiple circuits comprising distinct molecularly defined neuron populations (45, 46). These networks integrate the peripheral signals of the metabolic and visceral state (e.g., hormones and metabolites) and either coordinate or suppress behavioral responses in seeking and consuming food. The brain’s central nervous system (CNS) controls food intake and energy expenditure through tight coordination between multiple neuronal populations (47). AgRP neurons are the most important distinct neuronal population existing in the arcuate nucleus of the hypothalamus (ARH), accompanied by POMC neurons, which perform the opposite function in the anorexigenic (appetite-suppressing) ability (46, 48). The coordinated regulation of the neuronal circuit that involves these neurons is essential in maintaining energy balance. Any disturbance of these pathways may result in hyperphagia/obesity or hypophagia/starvation (8, 43). In fish, as in all vertebrates, the brain is also the major regulatory center for regulating food intake. Neuropeptides derived from the hypothalamus regulate food intake by stimulating (anorexigenic factors) or suppressing (anorexigenic factors) appetite (49).

The hypothalamus is a key brain region that regulates homeostasis, such as stress responses, sexual behavior, and energy homeostasis (50). The ARH area of the hypothalamus, located next to the third ventricle, is undoubtedly one of the best-characterized brain regions and is related to the control of feeding behavior (8). AgRP neurons are present in this region and are well-positioned with the POMC neurons located in the hypothalamus, receiving information from peripheral organs (50). The ARH resides in the ventromedial nucleus of the hypothalamus, which receives a rich blood supply due to its proximity to the median eminence, a site characterized by an incomplete blood–brain barrier (BBB) (51). Thus, body substance and energy status information from peripheral organs easily accesses these areas of neurons. In addition, this area receives intensive input from multiple parts of the central nervous system (52). Therefore, AgRP neurons are in an optimal position to integrate peripheral and central inputs to produce a central command for feeding behavior. Furthermore, the activity of the neurons is modulated by multiple neurotransmitters and/or hormones (23, 33, 39).

Agouti-related protein or peptide is secreted and released by the AgRP neurons (7). Studies that focused on Agouti-related protein neurons have identified multiple circuit elements that can elicit or suppress feeding behavior and have found that the AgRP peptide is not the only peptide produced by the neurons; NPY is also co-expressed in the same neurons (53–55). Therefore, the neurons are more frequently called NPY/AgRP neurons. Whereas AgRP cell bodies are restricted to the ARH, AgRP neuron synapses project to multiple areas of the hypothalamus and brain, and all AgRP terminals contain NPY (53). AgRP and NPY have a close functional relationship for the expression of these peptides and are similarly modulated under various physiological conditions. The activity of NPY/AgRP neurons is excited by orexigens, such as ghrelin and ORX, and decreased by leptin. GABA is the third bio-functionally released factor by AgRP neurons (56, 57). Numerous studies have shown that in AgRP neurons, NPY and GABA mediate acute feeding (55). The neuropeptide AgRP and its interaction with melanocortin receptors are involved in the long-term regulation of feeding behavior (58).

**Figure 1:**
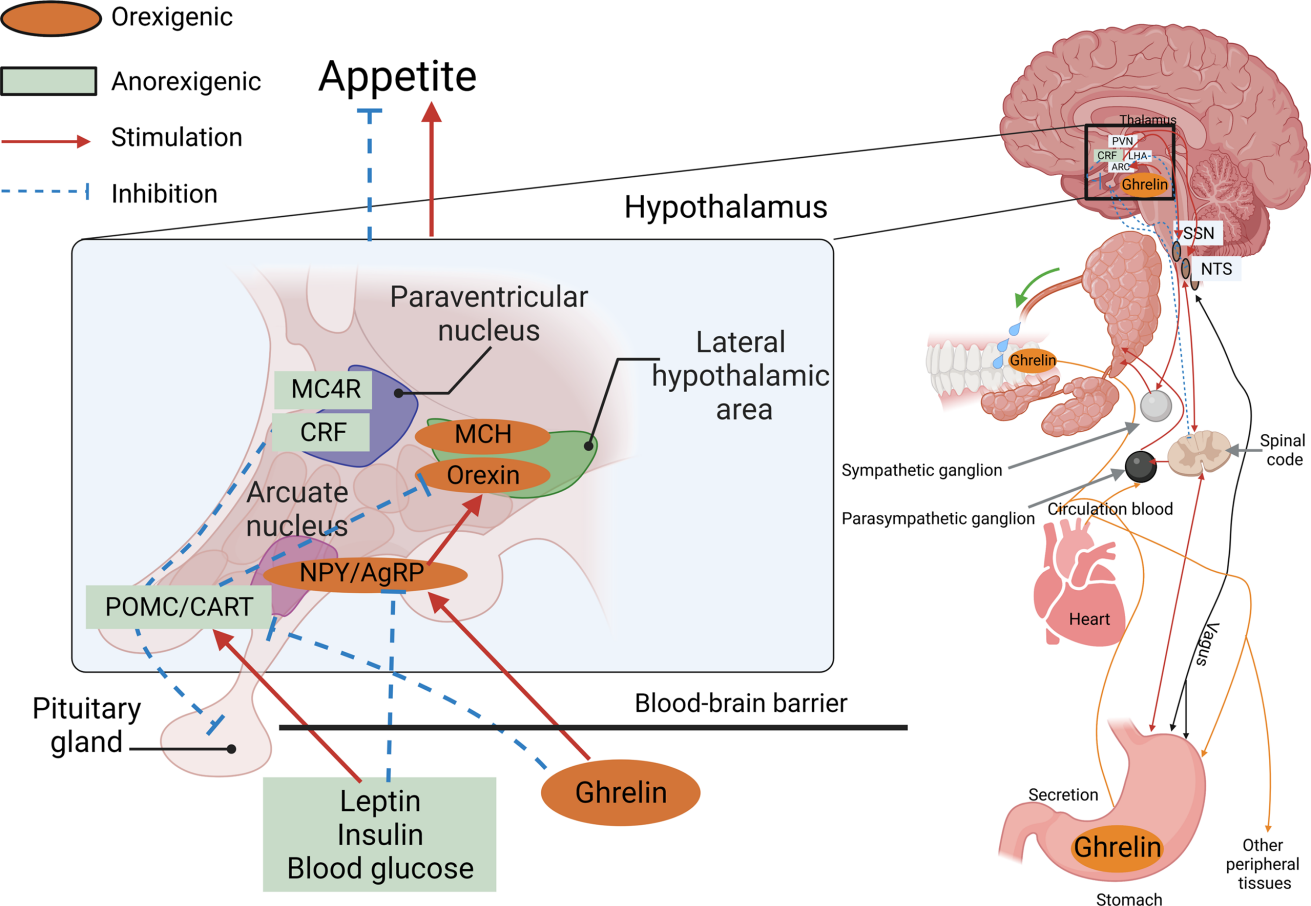
AGRP neuropeptide is released by AgRP neurons. An AgRP neuron’s location within the arcuate nucleus of the hypothalamus, co-positioned with POMC neurons located in the median eminence, and an incomplete blood–brain barrier (BBB) receive information from the peripheral organs.

The physiological mechanisms controlling appetite in vertebrates are relatively conserved, and many of the neuropeptides and hormones involved in central appetite regulation in mammals are also found in fish. The central signals produced by the hypothalamus are essential for the control of food intake, and so far, the major hormones and neuropeptides described in teleost fish, and their possible involvement in the central control mechanisms of appetite, have been conserved in a similar way as in mammals (49). In summary, as shown in **Figure 1**, the AGRP neuropeptide is released by AgRP neurons, which are well-positioned in the median eminence of the arcuate nucleus in the hypothalamus within the central nervous system of the brain.

## AgRP in melanocortin system

Although the hypothalamus plays a key role in regulating food intake and glucose homeostasis, its specific signaling pathway was not clear until the discovery of the melanocortin system (59). The melanocortin system comprises anorexigenic POMC-expressing neurons and orexigenic AgRP-expressing neurons, melanocortin receptors (MCRs), and two types of endogenous ligands (60).

The MCRs include members of the MC1R-MC5R family, which are the products of a series of small genes and are the smallest known molecular weight subfamily of G protein-coupled receptors (GPCR) (61). All MCRs are highly homologous and share a common molecular structure that contains only one exon. However, their distribution and biological function vary (61, 62). MC4R is expressed in various regions of the central nervous system and is considered to be the most important receptor molecule for feeding regulation and energy metabolism, playing a key role in the control of appetite and body weight homeostasis (63).

In mammals, the two types of endogenous ligands for the MC4R receptors are activating and antagonistic ligands (63). Activating ligands include α-melanocyte-stimulating hormone (α-MSH), β-melanocyte-stimulating hormone (β-MSH), γ-melanocyte-stimulating hormone (γ-MSH), and adrenocorticotropic hormone (ACTH), collectively referred to as melanocorticoid (MC). All of these activating small peptide ligands are expressed by the *pomc* gene through differential splicing by POMC neurons that bind to MC4R, producing cell signals that inhibit feeding (64, 65). An antagonistic ligand is AgRP, which is expressed and secreted by AgRP neurons and associated with MC4R binding in order to produce appetite-promoting cellular signals (66).

**Figure 2:**
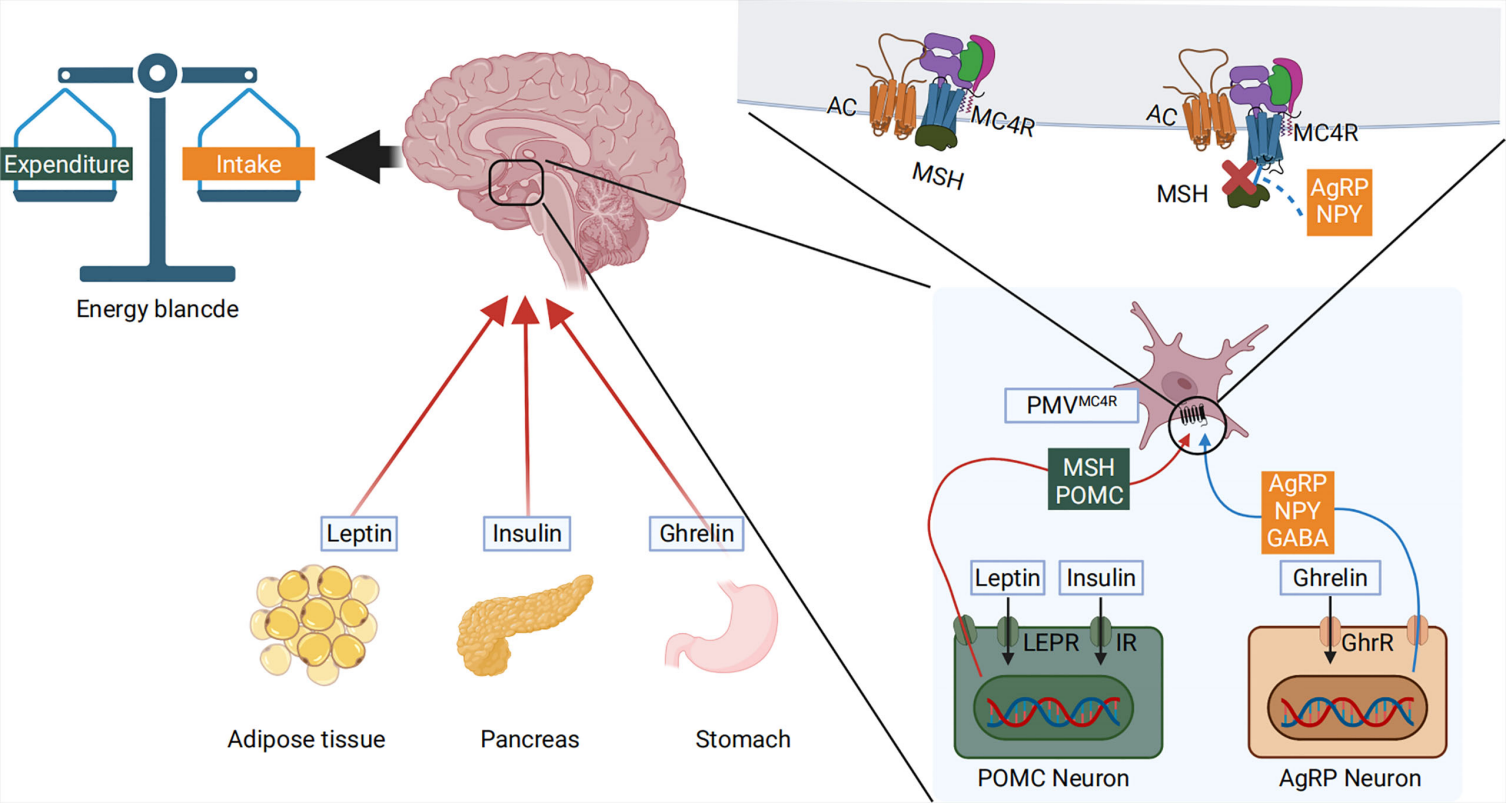
Peripheral blood nutrient concentrations and hormones constitute the peripheral energy state signals. High blood glucose levels cause pancreatic islets to produce insulin and adipose tissue to produce leptin. The low nutritional state and energy status allow the stomach to produce ghrelin. These molecules are transmitted to the hypothalamic center, where the melanocortin system responds to these signals. The LEPR and GHER receptors are expressed on the cell surface membrane of AgRP and POMC neurons. The hormones bind to their respective receptors, activating the *pomc* and *agrp* genes that are transcribed, producing α-MSH and AgRP. α-MSH further interacts with MC4R to reduce food intake. Conversely, when AgRP is produced, it directly blocks α-MSH mediated activation of the MC4R, thus inhibiting α-MSH’s action and increasing food intake.

**Figure 3:**
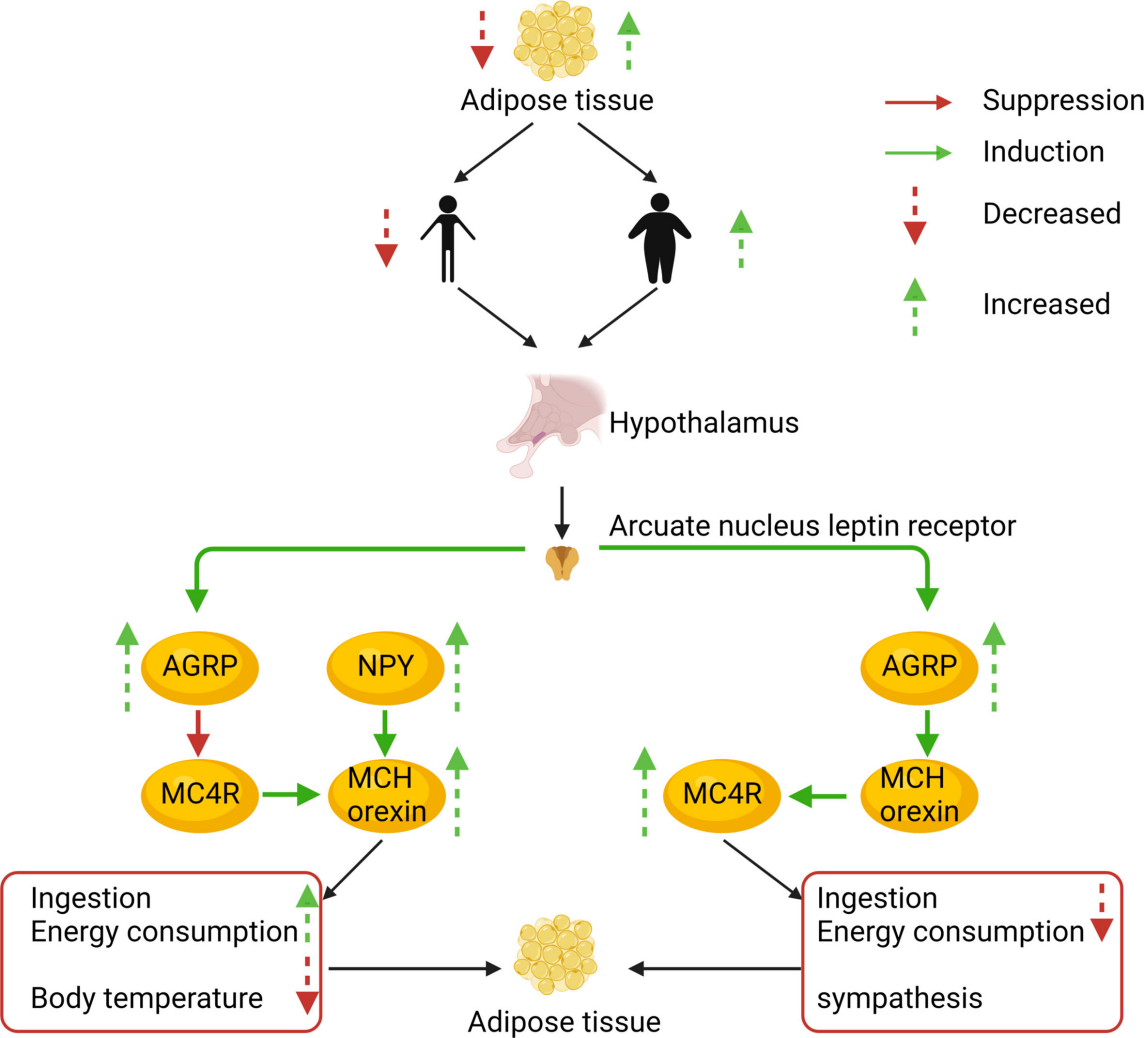
The AgRP and melanocortin systems are also involved in body weight regulation. To illustrate the prevalence of obesity, increased research efforts are underway to define how peripheral hormones and metabolites regulate energy homeostasis. The melanocortin system is crucial for normal energy homeostasis both in rodents and humans. They are regulated by peripheral hormones such as leptin and insulin as well as nutrients such as glucose, amino acids, and fatty acids.

Gastrointestinal glucose concentrations are translated into a signal that the melanocortin system can sense. Peripheral blood nutrient concentrations and hormones, combined with afferent neurons, constitute the peripheral energy state signal system (67). Nutrient intake and component content are immediately sensed in the periphery. They are then converted into gut-derived humoral and neural signals that are transmitted to the hypothalamic center, where the melanocortin system, the AgRP neurons, and the POMC neurons respond to these signals (68). Using leptin and ghrelin as an example, peripheral leptin and ghrelin are the first response signals of appetite suppression and enhancement, respectively (69). Leptin is a 16kD protein encoded by the obesity genes (*obese, ob*) and is primarily produced and secreted by adipocytes (70). Ghrelin is a gastrointestinal hormone produced by epithelial cells lining the stomach’s fundus and is now recognized as the only hormone to stimulate food intake peripherally (71). When peripheral energy deficits are integrated with high leptin signals, leptin will bind to its receptor LEPR on hypothalamic neurons, activating the intracellular energy sensor mTOR. The mTOR energy sensor then regulates the expression of downstream POMC neurons by acting on its effector S6K1, releasing the appetite-related peptide α-MSH, interacting with MC4R, and finally reducing feeding behavior (72, 73). In contrast, when peripheral energy is in surplus, ghrelin performs the opposite function through a similar process. Ghrelin’s receptor in hypothalamic neuron cells is GHER. Downstream, ghrelin activates AgRP neurons, promoting AgRP neuropeptides to block α-MSH’s binding to MC4R (74–76). The Gut–Brain Cross-Talk (77), Gut–liver axis (78), and the microbiota–gut–brain axis (MGB) (79) all of these pathways play important roles during this process. AgRP, the melanocortin system, and nutrient signal transduction are shown in **Figure 2**. AgRP and the melanocortin system are involved in body weight regulation as described in **Figure 3**.

## AgRP in fish appetite regulation and glucose sensing

Integrated nutrients play a vital role in the aquaculture industry. Carbohydrates, such as starch, are the most economical source of feedstock to provide energy. It is generally believed in the industry that low starch (below 8%) can be the breakthrough for carnivorous fish feed. Since 2017, the low starch feed has become the biggest growth point of high-grade extrudate feed in the industry. But carnivorous species have a limited capability of using carbohydrates as energy sources (80). The poor adaptation to high dietary carbohydrate loads in carnivorous fish may induce glycogen accumulation in liver tissue and further initiate glucose and lipid metabolic disorders in fish (81–83). On the other hand, protein nutrients are not only one of the most important nutrients in fish feed but also the most expensive supplements in fish feed (84). Fishmeal is the most commonly used protein source in fish farming (85). Even today, fishmeal is still a highly desirable protein source in aquaculture feed due to its high protein level, high digestibility, and ideal nutrient profile (86). However, fishmeal derived from wild-captured forage fish is costly and unsustainable because of its high demand and limited resources, have led to economic and ecological challenges (87, 88). Various plant-based ingredients, such as by-product meals from oilseeds, including soy, cotton, and rape seeds, have been used as fishmeal alternatives in aqua feed due to their relatively high protein concentration, stable production, low nitrogen and carbon emissions, and lower market price (89–91). However, there were several obvious unpleasant phenotypes when starch and plant protein were used in carnivorous species’ feed (92).

**Figure 4:**
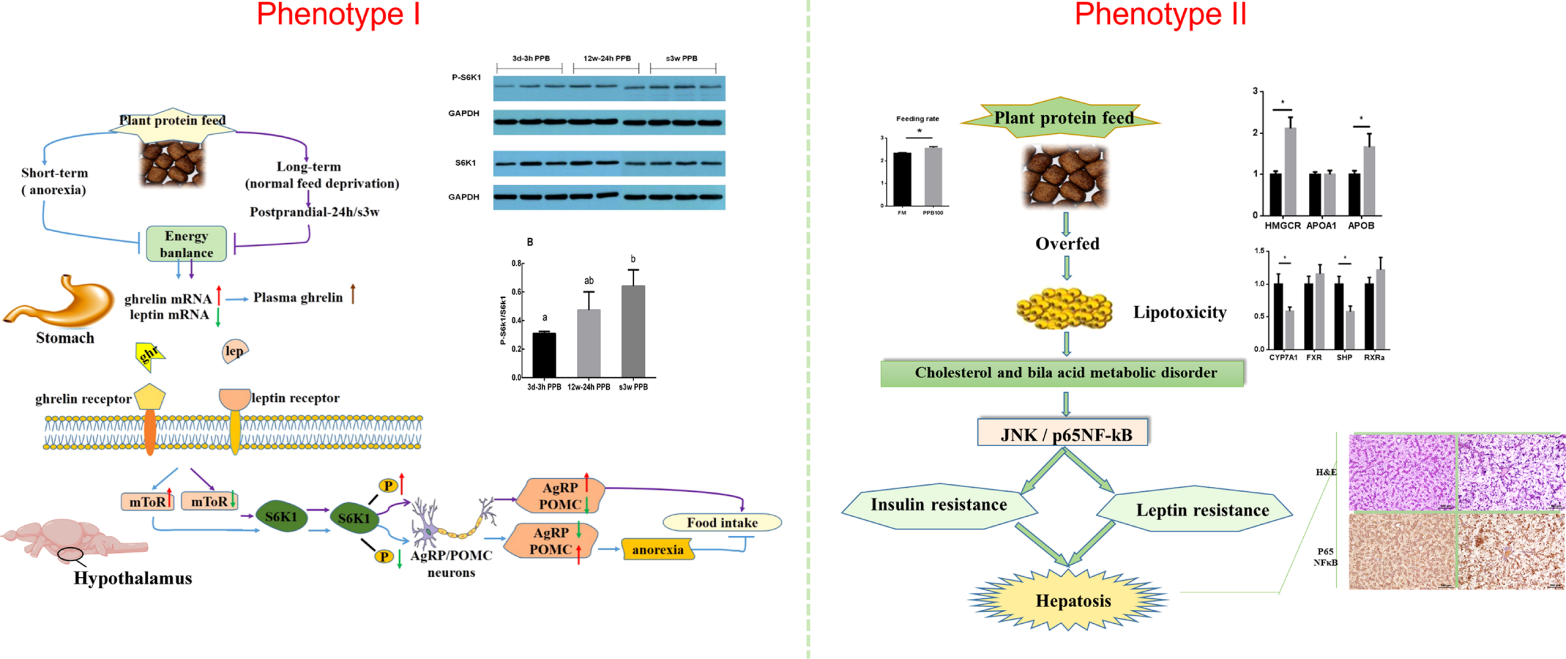
Two phenotypes of Japanese seabass when fishmeal is replaced by plant protein. Phenotype one causes obvious feeding inhibition, significantly reduced growth performance and high mortality rates. Phenotype two is normal feeding and survival, but the fish gradually experience protein, glucose, and lipid metabolism disorders, followed by chronic inflammation, hepatocyte apoptosis, and liver damage.

Feeding carbohydrate or plant protein feed added to carnivorous fish often induces feeding inhibition, resulting in a significant decrease in the feeding rate, thereby reducing growth performance (92, 93). Our lab has been researching aquatic feed for over 10 years (94, 95). We found that using plant protein instead of fish meal causes two phenotypes in Japanese seabass (*Lateolabrax maculatus*) (96, 97). Feeding inhibition significantly reduces growth performance and causes high mortality rates (96). The other is normal feeding and survival. However, the fish gradually experience protein, glucose, and lipid metabolism disorders, followed by chronic inflammation, hepatocyte apoptosis, and liver damage (97). The two phenotypes are shown in **Figure 4**.

In order to solve this basic research topic of industrial and fish feed applications, a series of research on plant protein replacement has been performed regarding aquatic nutrition. This area has made significant progress, making it one of the most important and actively researched fields concerning aquatic nutrition and metabolic regulation (93, 98–102). Scientists have tried to answer the questions surrounding feeding inhibition and nutrient metabolism disorders of carnivorous fish after replacing fish meal with plant protein by studying the raw ingredients of plant protein (amino acid pattern, carbohydrates, anti-nutritional factors), fish nutrition sensors, and fish nutrition response effectors (103). These studies highlight how fish process nutrient information, provide signal feedback, and issue instructions and behavioral responses in response to plant protein diets (104). In Japanese seabass, when receiving a diet high in plant protein content, an extreme anorectic response in the short term, followed by feeding adaptation and eventual compensatory intake after long-term plant protein feeding, was observed (89, 98, 101). Adaptation occurred within 8 to 12 weeks depending on the type of plant protein (85, 95). Anorexia was the main reason for poor growth performance and higher mortality rates in response to plant proteins for Japanese seabass during the first stage. For carnivorous fish, one of the reasons for perch anorexia due to plant protein may be due to the fact that plant proteins contain carbohydrates. Carnivorous fish have a poor ability to use carbohydrates, are intolerant to glucose, and cannot make good use of sugar as an energy source (105). Glucose metabolism disorder in Japanese seabass was expressed as uncontrollable fasting glycolysis and pyruvate aerobic oxidation 24 h postprandial with significantly upregulated glucose kinase (GK), pyruvate kinase (PK), and pyruvate dehydrogenase (PDH) gene expression, which potentially over-produces acetyl-CoA as the substrate for protein and lipid synthesis (97).

**Figure 5:**
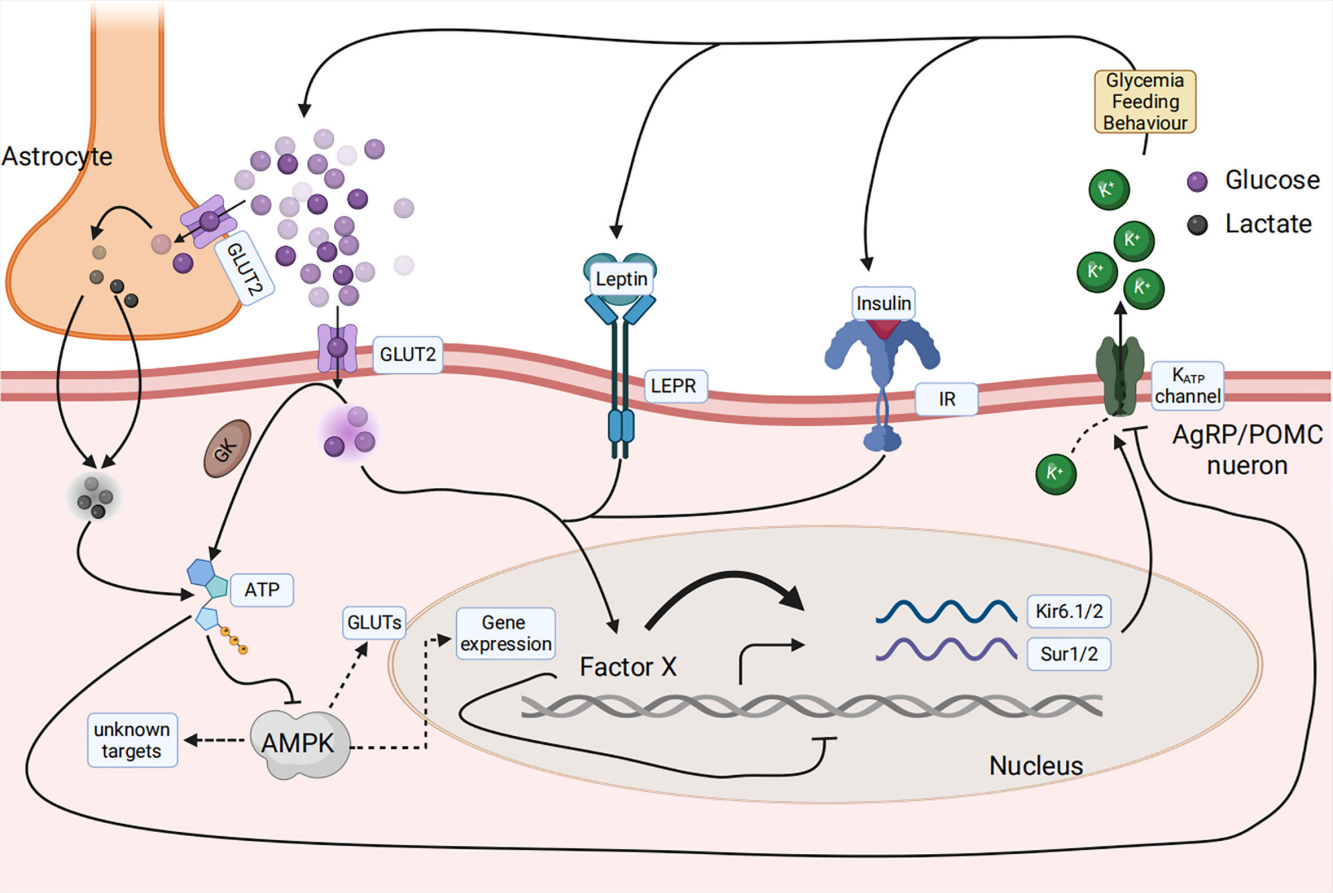
Glucose sensing and response in the arcuate nucleus in the hypothalamus of the central nervous system senses the composition change of the feed. Glucose concentration signaling consisted of three signal types, including insulin, leptin, and blood glucose, that finally converged on AGRP or POMC neurons. Glucose can be directly taken up by the neuron *via* glucose transporters (GLUT), phosphorylated by glucose kinase (GK), and subsequently metabolized to generate ATP. Glucose can also be taken up by astrocytes, converted to lactate, and then transported to neurons, where it is used for ATP generation. ATP then binds to ATP-dependent potassium (KATP) channels and directly controls the electrical excitability of both POMC and AgRP neurons. AMPK is necessary for glucose sensing within these neurons.

The primary research question is: what is the main glucose sensor in the central nervous system used to sense the composition changes of the feed? The mRNA levels of the feeding regulatory genes in the periphery and central nervous system showed that AgRP is involved during the key points of the anorectic and adaptation stages (96). Glucose concentration signals are derived from three signal types: insulin, leptin, and blood glucose (15, 106). All signals finally converge on AGRP neurons in the central nervous system. The leptin and insulin signaling pathways have been discussed above. Glucose can directly reach neurons *via* the cerebrospinal fluid or a leaky blood–brain barrier and is then taken up by the neuron *via* glucose transporters (GLUT), such as GLUT2 on the cell surface membrane. Glucose is then phosphorylated by GK and is then metabolized to generate ATP or taken up by astrocytes, converted to lactate, and then transported to neurons, where it is used for ATP generation (107, 108). ATP binds to ATP-dependent potassium (K_ATP_) channels and directly controls the electrical excitability of both POMC and AgRP neurons (109). Conversely, ATP generation decreases the AMP/ATP ratio, leading to a decrease in AMPK activity. AMPK seems necessary for glucose sensing in POMC and AgRP neurons (110) (**Figure 5**).

## Conclusion

The relationship between different brain regions and biological behaviors has been studied for two to three hundred years (111). Today, we know that food consumption and energy homeostasis are controlled by the arcuate nucleus in the hypothalamus of the central nervous system of the brain, consisting of AgRP and POMC neurons, two endogenous ligands (AgRP, α-MSH), and melanocortin receptors (60). Many studies on obesity, anorexia, and metabolic disorders have explained the pathogenesis of appetite, obesity, and anorexia as well as their correlation to these neurons and signal perception systems (112). However, the knowledge available in fish about the hypothalamic integration of information about metabolic and endocrine changes in the expression of neuropeptides ultimately regulating food intake is limited (113–115). The mechanisms of glucose signaling and responses in mammals have been gradually elucidated during the past century. Within the field of aquaculture, carnivorous fish such as sea bass, when being fed starch added or plant protein to replace a fish meal for a more economical and sustainable feed, find that the fish develop anorexia and metabolic disorders due to carnivorous fish not being able to utilize carbohydrates. The carbohydrates are converted into glucose signals that can be transmitted to the hypothalamus. These signals inhibit the expression and release of the AgRP neuropeptide within AgRP neurons, thereby inhibiting the feeding behavior of fish and ultimately limiting their growth performance. In this study, AgRP neuropeptide in AgRP neurons was demonstrated to play a key role in the transition from the anorexia stage (plant protein feeding for 3 weeks) to the adaptation stage (feeding for 4–8 weeks). Furthermore, it is the gene with the most significant changes in gene expression (96, 115).

**Figure 6:**
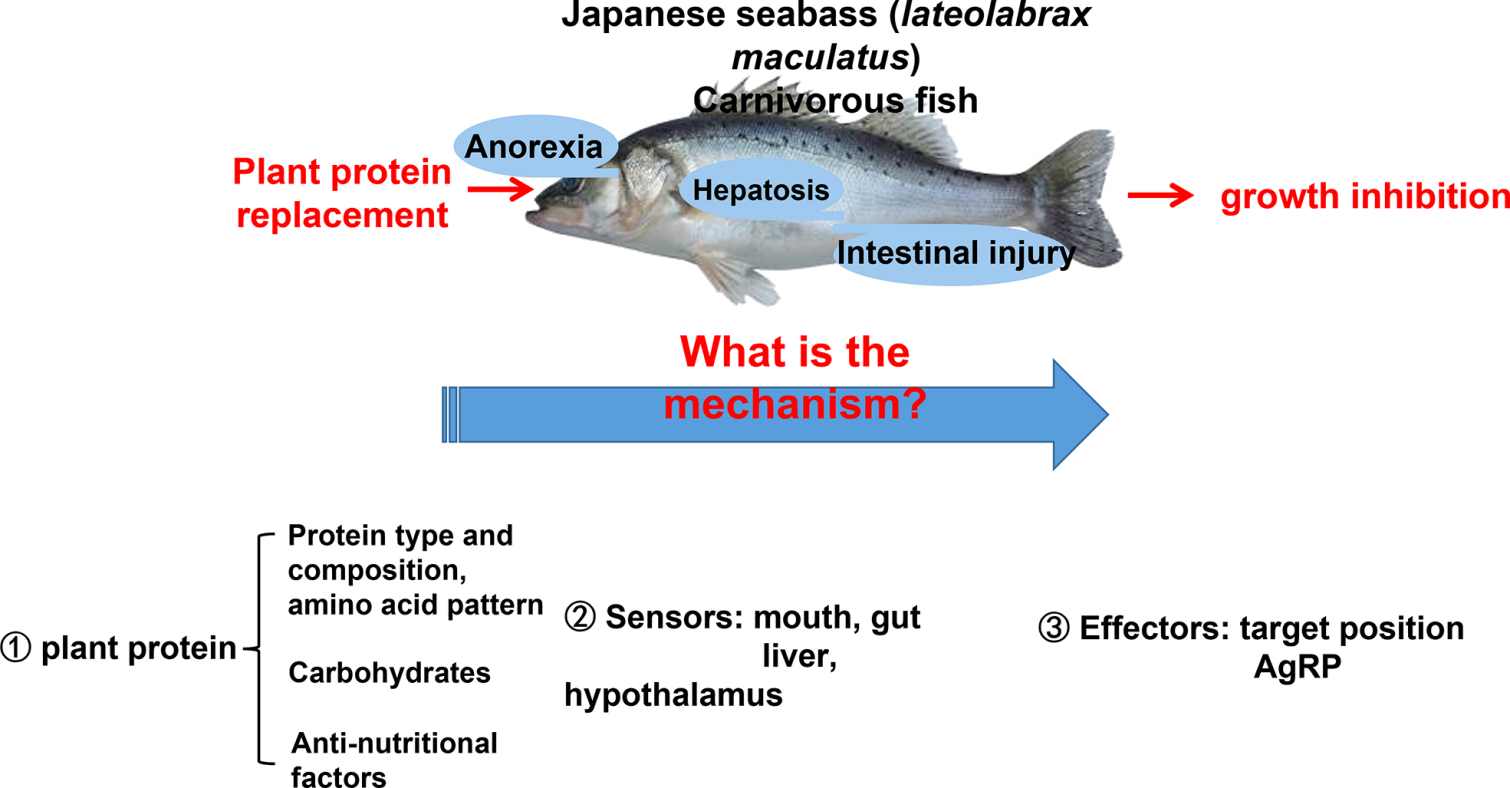
Research on the AgRP neuropeptide and its glucose sensor function will help to solve the efficient utilization of high-plant protein feed problems in carnivorous fish, thus promoting the development of aquaculture and the fish feed industry. This research clarifies the mechanisms underlying anorexia and metabolic disorders in carnivorous fish that affect their growth performance.

Next, an in-depth study of the neuropeptide AgRP and its interacting protein within the hypothalamus of fish, as well as the molecular mechanism of signal transduction during the regulation of feeding, revealed the key regulatory role of AgRP in the process of fish responding to plant protein replacement. The relevant regulatory networks will help solve the efficient utilization of high-plant protein feed in carnivorous fish, promoting the development of aquaculture and the feed industry, thus providing theoretical support for the selection and breeding of directional target species (116) (**Figure 6**).

## Author contributions

JH: conceptualization, writing—original draft, writing—review and editing, validation, visualization, supervision, project administration, and funding acquisition. XL: conceptualization, writing—review and editing, and project administration. YG: validation, writing—review and editing, and funding acquisition. XW: methodology, investigation, writing—original draft, and visualization. ZL: investigation, and discussion. TH: methodology, writing—review and editing. All authors contributed to the article and approved the submitted version.

## Funding

This study was supported by the National Natural Science Foundation of China (31802306) and the Agricultural Science and Technology Innovation Program (CAAS-ASTIP-2022-IFND).

## Conflict of interest

The authors declare that the research was conducted in the absence of any commercial or financial relationships that could be construed as a potential conflict of interest.

## Publisher’s note

All claims expressed in this article are solely those of the authors and do not necessarily represent those of their affiliated organizations, or those of the publisher, the editors and the reviewers. Any product that may be evaluated in this article, or claim that may be made by its manufacturer, is not guaranteed or endorsed by the publisher.
